# Broadness and specificity: ArdB, ArdA, and Ocr against various restriction-modification systems

**DOI:** 10.3389/fmicb.2023.1133144

**Published:** 2023-04-17

**Authors:** Anna A. Kudryavtseva, Eva Cséfalvay, Evgeniy Yu Gnuchikh, Darya D. Yanovskaya, Mikhail A. Skutel, Artem B. Isaev, Sergey V. Bazhenov, Anna A. Utkina, Ilya V. Manukhov

**Affiliations:** ^1^Laboratory for Molecular Genetics, Moscow Institute of Physics and Technology, Dolgoprudny, Russia; ^2^Laboratory of Structural Biology and Bioinformatics, Institute of Microbiology, Academy of Sciences of the Czech Republic, Nové Hrady, Czechia; ^3^Kurchatov Genomic Center, National Research Center Kurchatov Institute, Moscow, Russia; ^4^Center of Cellular and Molecular Biology, Skolkovo Institute of Science and Technology, Moscow, Russia; ^5^Laboratory for Microbiology, BIOTECH University, Moscow, Russia; ^6^Faculty of Physics, HSE University, Moscow, Russia

**Keywords:** ArdB, antirestriction, RMI, ArdA, conjugative plasmid

## Abstract

ArdB, ArdA, and Ocr proteins inhibit the endonuclease activity of the type I restriction-modification enzymes (RMI). In this study, we evaluated the ability of ArdB, ArdA, and Ocr to inhibit different subtypes of *Escherichia coli* RMI systems (IA, IB, and IC) as well as two *Bacillus licheniformis* RMI systems. Furthermore we explored, the antirestriction activity of ArdA, ArdB, and Ocr against a type III restriction-modification system (RMIII) EcoPI and BREX. We found that DNA-mimic proteins, ArdA and Ocr exhibit different inhibition activity, depending on which RM system tested. This effect might be linked to the DNA mimicry nature of these proteins. In theory, DNA-mimic might competitively inhibit any DNA-binding proteins; however, the efficiency of inhibition depend on the ability to imitate the recognition site in DNA or its preferred conformation. In contrast, ArdB protein with an undescribed mechanism of action, demonstrated greater versatility against various RMI systems and provided similar antirestriction efficiency regardless of the recognition site. However, ArdB protein could not affect restriction systems that are radically different from the RMI such as BREX or RMIII. Thus, we assume that the structure of DNA-mimic proteins allows for selective inhibition of any DNA-binding proteins depending on the recognition site. In contrast, ArdB-like proteins inhibit RMI systems independently of the DNA recognition site.

## Introduction

Antirestriction genes are typically found in various mobile genetic elements such as conjugative plasmids, transposons, or bacteriophages. Antirestriction proteins, ArdA, ArdB, and Ocr, inhibit type I restriction-modification enzymes (Krüger et al., 1982; Delver et al., 1991) and have no effect on restriction-modification enzymes of type II (Belogurov et al., 1985).

DNA-mimic ArdA-type antirestriction proteins (including the Ocr protein from the T7 phage) are known to be efficient against the both restriction and modification activities of RMI since they are able to interact with HsdM_2_HsdS_1_ and HsdR_2_HsdM_2_HsdS_1_ complexes (McMahon et al., 2009). Unlike ArdA, the antirestriction mechanism of ArdB remains a mystery, at least it is clear that it is not related to DNA mimicry (Serfiotis-Mitsa et al., 2009). Antirestriction proteins of the ArdB family inhibit anti-phage defense of the type I restriction-modification enzymes (RMI) *in vivo* (Belogurov et al., 1993) and do not interact with assembled RMI complexes *in vitro* (Serfiotis-Mitsa et al., 2009). In our previous studies, we hypothesized that ArdB protein blocks the translocation of restriction complex along unmodified DNA through interaction with the R-subunit of the type I restriction-modification enzyme (Balabanov et al., 2019; Kudryavtseva et al., 2020). This assumption was indirectly confirmed by the interaction of ArdB with DNA in *E. coli* cells (Kudryavtseva et al., 2020).

While *in vitro* ArdB was shown not to affect EcoKI restriction, it was found to have antirestriction activity *in vivo* against different families of RMI systems, including IA (EcoKI), IB (EcoA), IC (EcoR124), and ID (StySBLI) (Belogurov et al., 1993; Serfiotis-Mitsa et al., 2009).

In this study, the antirestriction activity of ArdB from conjugative plasmid R64 was evaluated against the IA–IC RMI families *in vivo* and *in vitro*. *In vivo* experiments were performed in the same expression system for all three families, which made it possible to directly compare the antirestriction activity of ArdB. Additionally, we investigated the versatility and specificity of ArdB, ArdA (pKM101 and ColIb-P9), and Ocr by testing the interaction with RMI systems of gram-positive bacteria *B. licheniformis*, RMIII system EcoPI, and BREX system from *E. coli*.

## Methods

### Strains and cultivation conditions

Strains *E. coli* TG1 (K-12 *glnV44 thi-1* Δ(*lac-proAB*) Δ(*mcrB-hsdSM*)5(*rK–mK–*) F′ [*traD36 proAB*+ *lacIq lacZ*Δ*M15*]); *E. coli* AB1157 (*thr-1 ltu-6 proA2 his-4 argE3 thi-1 lacY1 galK2 ara14 mtl-1 xyl-5 tsx-33 rpsL31 supE44, rk*+ *mk*+); and *E. coli* BL21 (DE3) (*F– ompT gal dcm lon hsdSB(rB –mB –)* λ*(DE3 [lacI lacUV5-T7p07 ind1 sam7 nin5])* [*malB*+] were obtained from VKPM (Russian National Collection of Industrial Microorganisms, Moscow).

*E. coli* strain TG1 has neither RMI nor RMIV systems. The genotype of TG1 allows us to use it in our studies and to be sure that there are no additional restriction or modification processes (Supplementary Table 1).

Bacteria were grown in flasks (150 ml) of LB medium on a shaker (New Brunswick Scientific, USA) at 37°C and 200 rpm. Solid medium was prepared with 1.5% agar. Transformed cells were grown in the medium containing 100 μg/ml of ampicillin, 30 μg/ml of chloramphenicol, or 20 μg/ml of kanamycin.

The optical density (OD) of bacterial suspension was measured at 590 nm with a KFK-2MP photocolorimeter (ProfMT, Russia).

### Plasmids

DNA cleavage with restriction endonucleases, ligation of DNA fragments, electrophoresis in agarose gel, and isolation of DNA fragments from agarose gel were performed according to standard techniques (Wood, 1983).

Transmissible R64 (IncI1) plasmid (GenBank AP005147) was used as a source of the *ardB* gene (*yfeB* in R64).

*B. licheniformis* DSM13 (NCBI NC_006322.1) strain from VKPM was used as a source of a gene of the RMI system of gram-positive bacteria. *B. licheniformis* DSM13 strain contains at least two predicted clusters of RMI genes with the following coordinates: 749489-756576 (named BlihIA) and 4163454-4171190 (named BlihIB). These systems were cloned into the pIRal vector (Bazhenov et al., 2023) using the EcoRV site, and primers are listed in Supplementary Table 2. The final constructs were named pIRal-2_RM-Ia and pIRal-2_RM-Ib, respectively.

To make pArdBRham compatible with the pBTB-2 vector expressing BREX and EcoPI systems, the antibiotic resistance gene was exchanged for *bla*, amplified from the pBAD vector, and the final construct was obtained by Gibson Assembly with NEBuilder HiFi master mix (NEB).

EcoPI *mod* and *res* genes together with the 120 bp upstream region containing the native promoter were cloned into pBTB-2 using P1 phage genomic DNA as a matrix [reference strain c1.100 (Łobocka et al., 2004)]. The final plasmid was assembled from the two PCR fragments by Gibson Assembly with NEBuilder HiFi mastermix (NEB), and the *araC* gene was removed from the pBTB-2 backbone in the process (primers listed in Supplementary Table 3).

The constructs used are presented in Table 1.

**Table 1:** Plasmids used in the present study.

**Plasmid**	**Description**	**Source**
pACYCEcoKI	Vector pACYC184, contains genes, which encode IA RMI-system EcoKI. Cm^r^.	Kudryavtseva et al., 2023
pAM35	Vector pACYC184, contains genes, which encode IB RMI-system EcoAI. Cm^r^.	Kudryavtseva et al., 2023
pKF650	Vector pACYC184, contains genes, which encode IC RMI-system EcoR124II. Cm^r^.	Patel et al., 1992
pIRal-2_RM-Ib	Vector pIRal, contains genes, which encode RMI-system a from *B. licheniformis*. Km^r^.	This work
pIRal-2_RM-Ib	Vector pIRal, contains genes, which encode RMI-system b from *B. licheniformis*. Km^r^.	This work
pVMC3	Plasmid contains genes, which encode IA RMI-system EcoKI. Ap^r^, ori ColEI.	Weiserova et al., 1993
pBREX AL	Type I BREX HS cluster cloned in pBTB-2, Km^r^.	Gordeeva et al., 2019
pBTB_EcoPI	EcoPI RMIII defense system of the phage P1 cloned into pBTB-2, Km^r^.	This work
pArdBRham	Vector pRhamhIL-10LT (NCBI txid1873718), contains the gene, which encodes ArdB(R64) under the PrhaB promoter. Km^r^.	Kudryavtseva et al., 2023
pArdBRham_AmpR	pArdBRham variant with Ap^r^.	This work
p15araArdB	Vector p15ara contains the gene, which encodes ArdB(R64) under the *L-araBAD* promoter. Cm^r^.	This work
pUCArdA(pSR3)	Vector pUC18 contains the gene, which encodes ArdA(pKM101) under the Plac promoter. Ap^r^.	Zavilgelsky et al., 2011
p15ara:ardA	Vector p15ara contains the gene, which encodes ArdA(ColIb-P9)under the *L-araBAD* promoter. Cm^r^.	Melkina et al., 2016
p15ara:ocr	Vector p15ara contains the gene, which encodes Ocr (T7 bacteriophage) under the *L-araBAD* promoter. Cm^r^.	Melkina et al., 2016
pBAD_Ocr	pBAD L24 vector encoding Ocr of the phage T7 under control of *L-araBAD* promoter, Ap^r^.	Isaev et al., 2020

### Antirestriction activity *in vivo*

Antirestriction activity of ArdB, ArdA, and Ocr proteins against IA, IB, IC RMI systems, BlihIA and BlihIB systems, and against BREX and EcoPI systems was measured using the efficiency of plaquing (EOP) assay with unmodified phage λ. The bacteriophage λ_vir_ was a kind gift from Prof. R. Devoret (France). Unmodified phage λ_0_ was grown on *E. coli* TG1. Modified λ_k_ phage was grown on *E. coli* AB1157 and used as a control.

The restriction and antirestriction values were estimated by comparing λ_0_ bacteriophage titers in *E. coli* K-12 TG1 cells carrying no plasmids with TG1 cells containing plasmids containing genes of restriction, antirestriction, or both.

The “double agar layer” method was used (Delver et al., 1991).

### Antirestriction activity *in vitro*

For the *in vitro* tests, proteins ArdB, EcoR124I, and EcoAI (M2SR2 complexes) were produced in BL21 (DE3) and JM109 (DE3) as described previously. HsdM_2_S and HsdR of EcoR124I were produced from plasmids pCOLAD-R124M_2_S (Taylor et al., 1992) and pTrcR124 (Janscak et al., 1996). HsdM_2_S and HsdR of EcoAI were overexpressed and purified from plasmids pJP21 and pJP22 (Janscak and Bickle, 1998).

ArdB was purified from PET-His-ArdB as described by Kudryavtseva et al. (2020) by using affinity chromatography (His-Trap, 5 ml, Cytiva) followed by gel filtration (S300 Sepharose FF, 1 x buffer NEB2, New England Biolabs).

After purification, all proteins were stored in 1xNEB2, 50% glycerol at −20 °C.

Antirestriction activity of ArdB proteins against IB and IC RMI-systems was studied *in vitro* as described in previous studies (Serfiotis-Mitsa et al., 2009; Csefalvay et al., 2015). Restriction enzymes EcoR124I and EcoAI were reconstituted *in vitro* from specific methyltransferases (M2S) and restriction subunits (HsdR) in a ratio of 1:6. Cleavage assays were done on circular DNA bearing one recognition site (Jindrova et al., 2005) for studied endonucleases. Reaction mixtures contained 10 nM DNA [circular plasmid pARK (Jindrova et al., 2005)], 10 nM endonuclease, and ± 300 nM of ArdB antirestriction protein. Reactions were initiated by the addition of ATP and S-adenosyl methionine to a final concentration of 2 mM and 0.2 mM, respectively and proceeded at 37°C for 20 min. The chosen ratio of HsdR to methylase has been experimentally determined as the ratio with maximum enzyme activity, and the further increase in HsdR concentration does not show enchanced activity. The total reaction volume was 100 μl. Aliquots of 10 μl were removed at the indicated time points in time-course experiments. The reaction was stopped by adding 0.25 volume of stop solution (3% SDS, 0.15M EDTA, 10% glycerol, and 0.1% bromophenol blue) and heating to 65°C for 5 min. Cleavage products were resolved on 1% (w/v) agarose gel and quantified in TotalLab Quant.

### Data processing

The efficiency of plaquing (EOP) was estimated as follows:


(1) $$\begin{array}{l} \text{EO}{\text{P}}_{\text{X}}=\frac{{\text{N}}_{\text{X}}}{{\text{N}}_{\text{TG}1}} \end{array}$$

where N_X_ is the number of λ_0_ phage plaques on the *E. coli* TG1 cells carrying gene “X” affecting the plaque forming and N_TG1_ is the number of λ_0_ phage plaques on *E. coli* TG1 (without any additional restriction or antirestriction genes).

To compare different endonucleases by their ability to limit λ_0_ growth on TG1 cell culture, the restriction coefficient (K_r_) was calculated as the ratio of “missed” plaques number to the number of plaques in the sample with endonuclease:


(2) $$K_{r}=\frac{{\text{N}}_{\text{TG1}}{-\text{ N}}_{\text{End}}}{{\text{N}}_{\text{End}}}$$

where N_End_ is a number of formed plaques on *E. coli* TG1 carrying specific endonuclease genes.

To compare different antirestriction proteins by their ability to alleviate restriction by different restriction endonucleases, the residual endonuclease activity (REA) coefficient was calculated. REA is normalized to K_r_ of corresponding endonuclease.


(3) $$REA=\frac{{\text{N}}_{\text{TG1}}{-\text{ N}}_{\left(\text{End}+\text{Ard}\right)}}{{\text{N}}_{\left(\text{End}+\text{Ard}\right)}}\;*\;\frac{1}{{\text{K}}_{\text{r}}}\;*\;100\%$$

where N_(End+Ard)_ is the number of formed plaques on *E. coli* TG1 carrying specific endonuclease and antirestriction genes. Putting (1), (2), and (3) together, we can rewrite the equation for RAE in terms of EOP as soon as N_End_ and N_(Ecnd+Ard)_ for each repeat are obtained in one experiment with common control N_TG1_:


(4) $$REA=\frac{{1-\text{EOP}}_{\left(\text{End}+\text{Ard}\right)}}{{\text{EOP}}_{\left(\text{End}+\text{Ard}\right)}}\;*\;\frac{{\text{EOP}}_{\left(\text{End}\right)}}{{1-\text{EOP}}_{\left(\text{End}\right)}}\;*\;100\%$$

Since EOP is at most 1 and EOP_(End)_ is lower than EOP_(End+Ard)_, REA takes a value between 0 and 100%. The average values of EOP and REA and their standard deviations were calculated for three independent experiment replications.

Diagrams for EOP and REA are obtained using the matplotlib utility (Droettboom et al., 2017).

## Results and discussion

### ArdB efficiently inhibits EcoKI, EcoR124, and EcoAI defense *in vivo*

**Figure 1:**
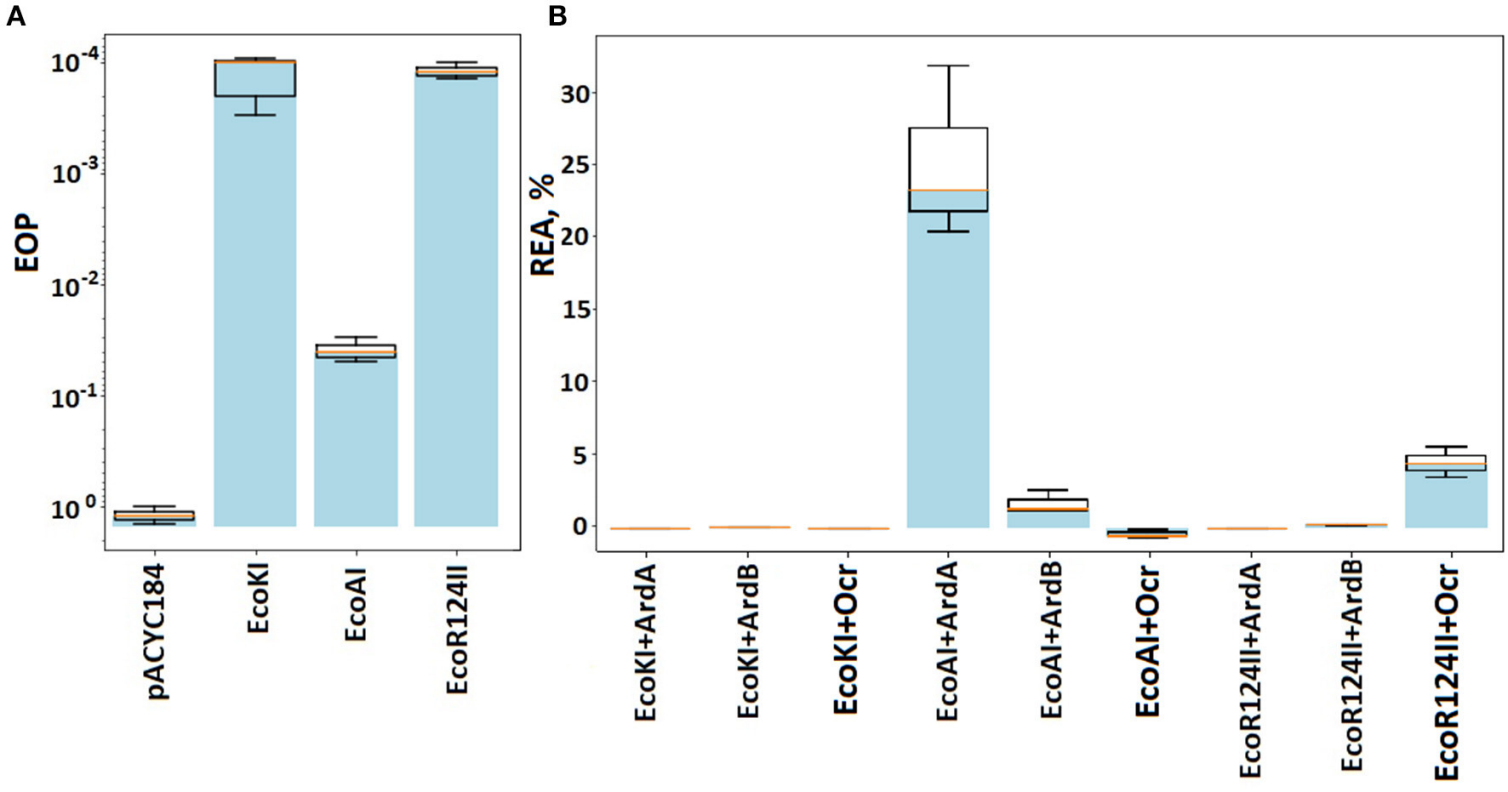
The efficiency of defense provided by RMI systems. Results of the λ_0_ phage plaquing (EOP) on a lawn of *E. coli* cells containing genes of various RMI systems of gram-negative bacteria. **(A)** EOP represents a ratio of a phage titer obtained on the experimental lawn relative to the TG1 lawn, which is sensitive to phage infection. Columns: pACYC184 – empty vector TG1pACYC184, EcoKI – TG1pACYCEcoKI, EcoAI – TG1pAM35, and EcoR124II – TG1pKF650. **(B)** Antirestriction activity of ArdB, ArdA, and Ocr. The residual activity of endonuclease (REA) represents the activity of endonuclease in the presence of ArdB, ArdA, or Ocr. Columns: EcoKI+ArdA – TG1pACYCEcoKIpUCArdA; EcoKI+ArdB – TG1pACYCEcoKIpArdBRam; EcoKI+Ocr – TG1pACYCEcoKIpBADOcr; EcoAI+ArdA – TG1pAM35pUCArdA; EcoAI+ArdB – TG1pAM35pArdBRam; EcoR124II+ArdB – TG1pKF650pArdBRam; EcoR124II+Ocr – TG1pKF650pBADOcr.

The antirestriction activity of ArdB(R64) against IA, IB, and IC RMI systems is presented in Figure 1, Supplementary Table 4.

The presence of EcoKI and EcoR124 systems reduced phage λ_0_ plaquing abilities by four orders of magnitude, while EcoAI system by ~300. ArdB expression (pArdBRam, PrhaB promoter) resulted in complete inhibition of EcoKI defense and improved phage plaquing by 2.5 orders of magnitude on the EcoR124 lawn. These results were obtained without the addition of the inducer due to promoter leakage, while induction of the rhamnose promoter did not lead to a significant improvement in phage seeding (data not shown). Apparently, the ArdB protein aggregates in cells when high concentrations are reached (Kudryavtseva et al., 2023). The antirestriction effect of ArdB against EcoKI and EcoR124 appears to be quite similar. The number of recognition sites in λ phage genome could explain the difference in phage plaquing: five for EcoKI and 12 for EcoR124. Even a low concentration of ArdA protein from pKM101 (pUCArdA, Plac promoter) completely restored the λ_0_ phage plaquing efficiency for both EcoKI and EcoR124.

ArdB (pArdBRam and PrhaB promoter) demonstrated more than one order of magnitude antirestriction effect against EcoAI. Despite the low-level restriction activity of IB RMI-system EcoAI, it was not possible to obtain a significant antirestriction effect of the ArdA protein from pKM101 (pUCArdA and Plac promoter). It should be noted that all our attempts to increase ArdA or Ocr concentration in the cell using stronger promoters or induction failed due to the high toxicity of these proteins (data not shown). Herein, the highest possible “effective” concentration was used for all three tested antirestriction proteins.

The fact must be taken into account that another DNA-mimic protein Ocr successfully inhibited all three RMI-systems from *E. coli*.

### *In vitro* antirestriction activity of ArdB against EcoR124 and EcoAI

Antirestriction activity of the ArdB against IC and IB RMI-system was estimated *in vitro* using the plasmid pARK (Jindrova et al., 2005), which contains a single EcoR124 and EcoAI recognition site. Unlike the *in vivo*, which contained EcoR124II, in this assay the EcoR124I enzyme was used. Enzymes EcoR124I and EcoR124II are allelic. The only difference between them is four amino acids in the recognition site of the HsdS subunit (Price et al., 1989; Gubler and Bickle, 1991). EcoR124I has 14 recognition sites in λ phage, while EcoR124II has 15 recognition sites in λ phage. ArdB's activity seems to be independent of the recognition site; therefore, it is possible to use EcoR124I here. For the *in vitro* tests, proteins ArdB, EcoR124I, and EcoAI (M2SR2 complexes) were isolated from BL21 (DE3). Purified ArdB was tested to inhibit pARK plasmid restriction by EcoR124 and EcoAI *in vitro* with periodic sampling.

**Figure 2:**
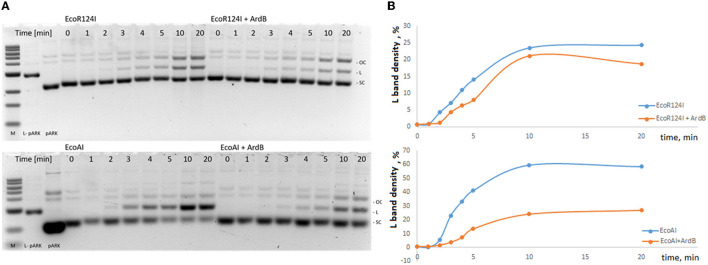
Influence of ArdB presence on the endonuclease activity of IB and IC RMI-systems EcoR124I and EcoAI *in vitro*. **(A)** 1% agarose gel electrophoresis: lane 1-−1kb ladder, lane 2—linear plasmid pARK, lane 3—circular plasmid pARK, lanes 4–11—incubation time (min) of the mix containing 10 nM pARK, 10nM EcoR124I, or EcoAI endonuclease; lanes 12–19—incubation time (min) of the mix containing 10 nM pARK, 10nM EcoR124I, or EcoAI endonuclease with 300 nM ArdB). **(B)** The percentage of linear plasmid at total plasmid band density (L band density, Lbd) measured with TotalLab Quant software. A typical experiment is presented. The ratios Lbd(EcoR124I)/Lbd(EcoR124I+ArdB) and Lbd(EcoAI24I)/Lbd(EcoAI24I+ArdB) were measured for three independent experiments at 5 min and equaled 1,35±0,1 and 3,38±0,3, respectively.

The restriction activity of both EcoAI and EcoR124 in the assay was determined with the formation of linear plasmid DNA, which can be detected after 2 min of incubation (Figure 2). The addition of 0.3 mM ArdB to the incubation mix results in a delay in the linear DNA formation: Reliable detection of linear DNA occurs within a 3-min time point, and an overall decrease in restriction activity is noticeable at a lower concentration of the product (linear DNA) formation. This could indicate the decrease in the rate of the restriction and may point to the ability of the ArdB protein to inhibit the RMI complex *in vitro*. The effect is the same for both IB RMI-system EcoAI and IC EcoR124, which is in line with the *in vivo* experiments.

Previously, Belogurov's study reported that ArdB from PKM101 did not show an *in vitro* antirestriction effect, as well as ArdB's homolog KlcA which was studied by Dryden and colleagues. At the same time, it is important that both ArdB from PKM101 and KlcA from IncP-1b plasmid demonstrated antirestriction activity *in vivo* (Belogurov et al., 1993; Serfiotis-Mitsa et al., 2009). Here, we first showed the inhibition of the endonuclease activity by ArdB protein *in vitro;* however, it is still incomplete. This might indicate that an additional participant is necessary for an effective antirestriction activity of ArdB *in vitro*.

### ArdB inhibits RMI-systems from *B. licheniformis*

We've decided to investigate whether ArdA and ArdB proteins will demonstrate activity against distantly related RMI systems from gram-positive bacteria. For this reason, we analyzed the *B. licheniformis* genome and detected two RMI systems: 749489-756576 (named BlihIA) and 4163454-4171190 (named BlihIB). Detected genes were named *blihIA* and *blihIB* and cloned them into the pIRal vector for expression in *E. coli* cells.

*Escherichia coli* RMI systems were historically classified into subtypes IA, IB, IC, and ID according to the ability of genes of RMI systems to hybridize each other (Murray, 2000). *blihIA* and *blihIB* have no significant similarities in DNA sequences with any of the IA–ID representatives. Therefore, we cannot classify them into any IA–ID groups. Both BlihIA and BlihIB systems have about 20% percent of coverage with the IC EcoR124II system (Supplementary Figure 1).

**Figure 3:**
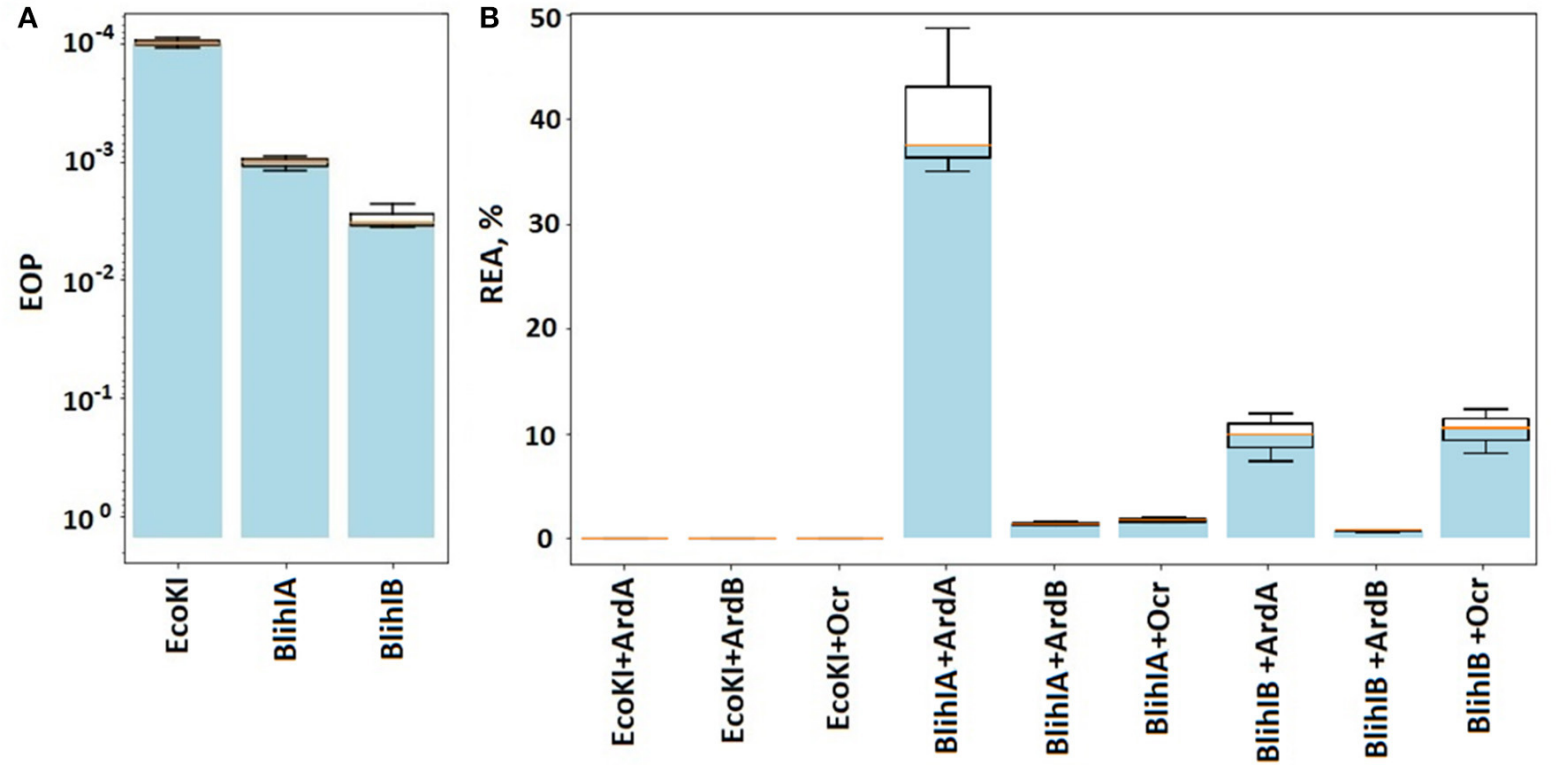
The efficiency of defense provided by RMI systems. **(A)** Results of the λ_0_ phage plaquing (EOP) on a lawn of *E. coli* cells containing genes of various RMI systems of gram-positive bacteria compared to the EcoKI system. Columns: EcoKI – TG1pVMC3, BlihIA – TG1pIRal-2_RM-Ia, and BlihIB – TG1pIRal-2_RM-Ib. **(B)** Antirestriction activity of ArdA, ArdB, and Ocr proteins. Residual endonuclease activity (REA) represents the activity of endonuclease in the presence of antirestriction proteins. Columns: EcoKI+ArdA – TG1pVMC3p15araArdA, EcoKI+ArdB – TG1pVMC3p15araArdB, EcoKI+Ocr – TG1pVMC3p15araOcr, BlihIA+ArdA – TG1pIRal-2_RM-Ia-p15araArdA, BlihIA+ArdB – TG1pIRal-2_RM-Ia – p15araArdB, BlihIA+Ocr – TG1pIRal-2_RM-Ia – p15araOcr, BlihIB+ArdA – TG1pIRal-2_RM-Ib – p15araArdA, BlihIB+ArdB – TG1pIRal-2_RM-Ib – p15araArdB, and BlihIB+Ocr – TG1pIRal-2_RM – Ib – p15araOcr.

Figure 3, Supplementary Table 6 shows the results of λ_0_ EOP assay in the presence of antirestriction genes (*ardB, ardA*, and *ocr*) and RMI-systems from *B. licheniformis*. First, we showed that both RMI systems cloned from the gram-positive *B. licheniformis* provide two orders of magnitude protection against λ phage when expressed in the heterologous *E. coli* system (Figure 3A). The restriction activity of BlihIA and BlihIB against λ phage in *E. coli* appeared to be lower than the classic EcoKI system.

Figure 3B shows that the ArdA, ArdB, or Ocr proteins, expressed from the p15ara vector, work with equal efficiency against the EcoKI system.

Thus, here we showed that all three antirestriction proteins ArdB, ArdA, and Ocr could inhibit gram-positive RMI-systems with varying efficiencies. DNA-mimic proteins ArdA and Ocr appeared to be less efficient against gram-positive RMI-systems compared to ArdB protein with the unknown antirestriction mechanism.

Moreover, DNA-mimic proteins demonstrated specificity once again, with different effects against BlihIA and BlihIB RMI systems.

### ArdA and ArdB antirestriction activity against BREX and RMIII defense systems

**Figure 4:**
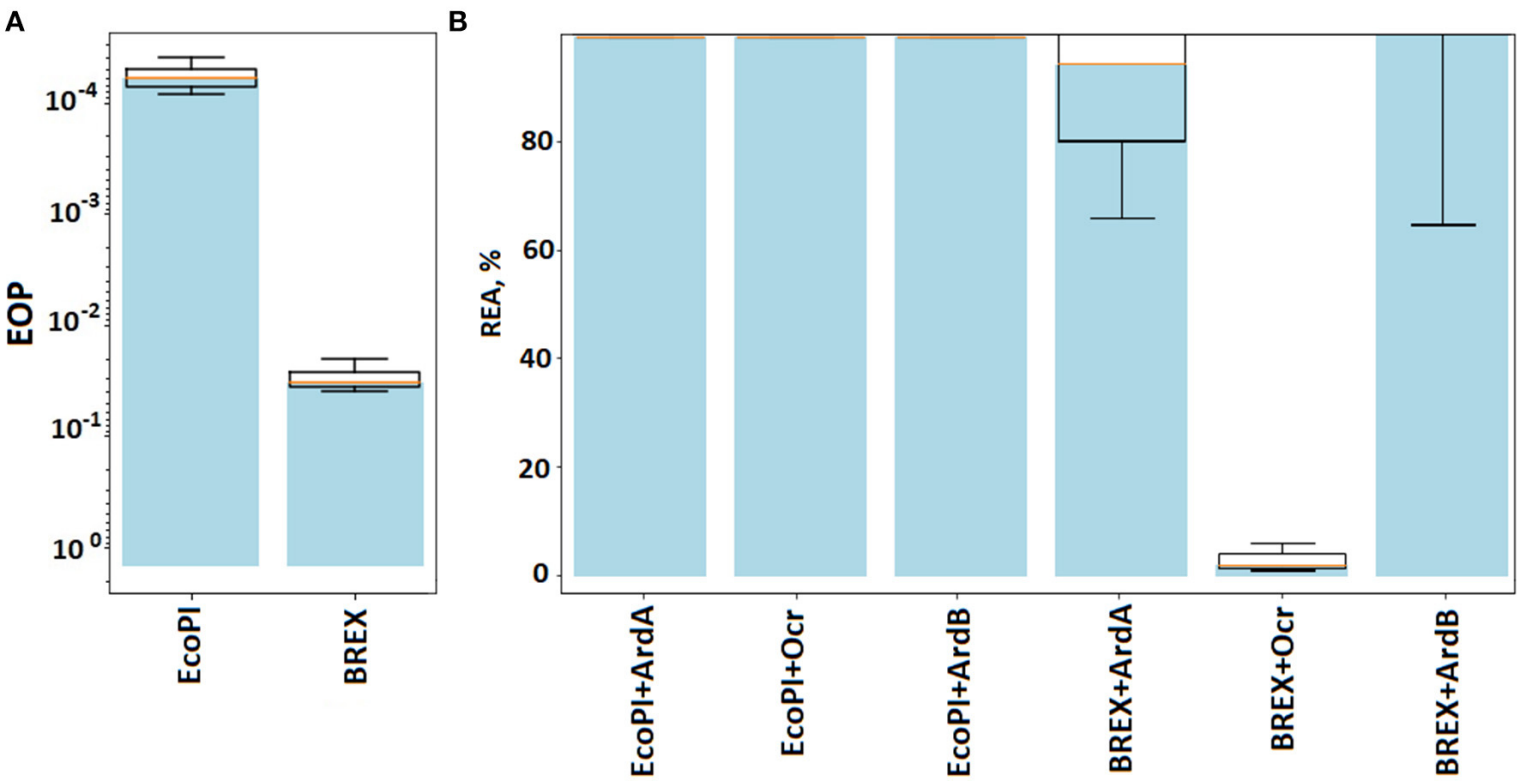
The efficiency of defense provided by BREX and RMIII (EcoPI) systems. **(A)** Results of the λ_0_ phage plaquing (EOP) on a lawn of *E. coli* cells containing BREX or RMIII (EcoPI) defense systems of gram-negative bacteria. Columns: EcoPI – TG1pBTB_EcoPI and BREX – TG1pBREX. **(B)** Antirestriction activity of ArdA, ArdB, and Ocr proteins. Residual endonuclease activity (REA) represents the activity of endonuclease in the presence of antirestriction proteins. Columns: EcoPI+ArdA – TG1pBTB_EcoPIpUCArdA(pSR3), EcoPI+ArdB(prha) – TG1pBTB_EcoPIpArdBRham_AmpR, EcoKI – TG1pACYCEcoKI, BREX + ArdA – TG1pBREXpUCArdA(pSR3), BREX + ArdB(prha) – TG1pBREXpArdBRam, EcoPI – TG1pBTB_EcoPI, EcoPI + Ocr – TG1pBTB_EcoPIp15ara:ocr, and BREX+Ocr – TG1pBREXp15ara:ocr.

In addition, we have decided to investigate whether ArdB or ArdA has the ability to inhibit two other defense systems: BREX (Goldfarb et al., 2015) and type III R-M EcoPI (Rao et al., 2014). A previous study showed that DNA-mimic Ocr (Isaev et al., 2020) and some other anti-RMI proteins (Andriianov et al., 2023) inhibit BREX defense, which means that BREX and RMI might share common structural or mechanistic features, despite the differences in their organization, so it was of interest to determine whether ArdA or ArdB has an anti-BREX activity. BREX provided two orders of magnitude defense against λ_0_ phage in the TG1 background, and defense was completely inhibited in the presence of the Ocr protein, as expected (Figure 4, Supplementary Table 7). The expression of ArdB and ArdA did not provided an anti-BREX effect (Figure 4), consistent with previous observations (Isaev et al., 2020). This result demonstrates that even at higher expression levels achieved from the p15ara vector, ArdA and ArdB do not interfere with the BREX function. The lack of ArdA anti-BREX activity is in contrast with the full BREX suppression by the DNA mimic - Ocr, which confirms that DNA-mimicry on its own is not sufficient for the inhibition of DNA-binding immunity systems.

Type III R-M system EcoPI from the phage P1, under the control of the natural promoter in the pBTB-2 vector, provided five orders of magnitude protection against λ_0_ phage in the TG1 strain (Figure 4). Neither ArdA nor the ArdB or Ocr expression had an effect on the EcoPI defense. Apparently, this is due to a principled difference in the structure of RMI and RMIII. Note that RMIII, like RMII (against which antirestriction enzymes do not work), does not contain an S-subunit.

The results highlight that DNA-mimic proteins Ocr and ArdA have different specificities against BREX and RMI defense, while ArdB most likely targets functions but not characteristic of BREX or RMIII.

## Conclusion

In this study, we compared the efficiency of the antirestriction protein ArdB against three subtypes of type I restriction-modification systems: EcoKI (IA), EcoAI (IB), and EcoR124 (IC) in similar expression conditions. It has been shown that the ArdB protein effectively inhibits the activity of all three restriction-modification systems.

The mechanism of the ArdB antirestriction activity remains a mystery, but our hypothesis of non-specific binding to DNA seems to be plausible in view of the data obtained in this study: The efficiency of ArdB inhibition seems not to depend on RMI complex recognition site specificity. Moreover, these results are also confirmed for the first studied RMI systems from gram-positive bacteria *B. licheniformis*. The results highlight the versatility and non-specificity of the antirestriction activity of ArdB to any RMI systems, regardless of the DNA recognition site. The inconsistent *in vitro* results of increased cleavage activity in the presence of ArdB may indicate the requirement of a “third player” of the antirestriction process that was absent in our *in vitro* system.

Affine purification of ArdB protein and concentration optimization of mixture components allowed us to demonstrate the ArdB antirestriction effect *in vitro*. The incompleteness of the *in vitro* effect may indicate the requirement of a “third player” of the antirestriction process which was absent in our *in vitro* system.

Earlier, we showed the direct interaction of ArdB with DNA (Kudryavtseva et al., 2020). The universality of ArdB's mechanism of activity against RMI-systems, its *in vitro* activity, and the lack of activity against BREX and Type III R-M indirectly point to possible mechanisms of antirestriction which were previously proposed (Balabanov et al., 2019). ArdB interacts with DNA and RMI-complex, and we propose that it might block the R-subunit translocation process—the stage characteristic of RMI systems but not of EcoPI or BREX systems.

On the contrary, the ArdA and Ocr proteins, despite the seemingly non-specific antirestriction mechanism relying on DNA mimicry, showed some selectivity to endonucleases, which recognize different DNA sites. It can be assumed that structurally different ArdA might have different specificities to the RMI system that they inhibit. For example, it is known that Acr DNA-mimic proteins show high specificity to CRISPR-Cas systems (León et al., 2021). We assume that ArdA-type proteins might also demonstrate a high degree of target specificity due to their structural features.

## Data availability statement

The original contributions presented in the study are included in the article/Supplementary material, further inquiries can be directed to the corresponding author.

## Author contributions

AK studied antirestriction activity against gram-negative and gram-positive RMI systems. AI, MS, and DY studied antirestriction activity against BREX and EcoPI. EG worked with gram-positive bacteria and searching RM-I genes in gram-positive bacteria. IM edited manuscript and organized work. SB performed data analysis. All authors contributed to the article and approved the submitted version.
