# Supplementary material for: Broadness and specificity: ArdB, ArdA, and Ocr against various restriction-modification systems
Source: Front Microbiol. 2023 Apr 17;14:1133144. doi: 10.3389/fmicb.2023.1133144 (PMC10149784; doi:10.3389/fmicb.2023.1133144)
Supplement: Supplementary file 1 [file Data_Sheet_1.docx]

Table S1. Results of the λ.0 and λ.k_(EcoAI)_ phage plaquing (EOP) on a lawn of *E. coli* cells TG1 and TG1 cells containing EcoAI genes. We actually have no evidence on mrr’s presence in our systems. To test the possible influence of *mrr* gene we used EcoAI RM-I system. EcoAI recognition site contains CTC(N6)CAGT sequence where CAG-site methylation could be inhibited by the *mrr* effect. We measured EOP for unmodified phage λ.0 and for EcoAI modified phage λ.k_(EcoAI)_.

|  | λ.0 | λ.k (EcoAI) |
| --- | --- | --- |
| TG1 | 1 | 1 |
| TG1pAM35(EcoAI) | 0,05±0,012* | 1±0,05 |

*the results for three independent experiments are presented

Table S2. Primers for *B. licheniformis* RMI-system cloning

| BlihIA_Dir | TCCGGGTAACGAATTCAAGCTTGATAAATACTCTATCGATGAGGGTGGGATATG |
| --- | --- |
| BlihIA_Rev | AGTCTGAGGCTCGTCCTGAATGATTAAATACCTTTTAAAAAATCCTCCGACTCTGACT |
| BlihIB_Dir | GTCTGAGGCTCGTCCTGAATGATACATTTGTTGCCATACTGGATTTGATGTCT |
| BlihIB_Rev | TCCGGGTAACGAATTCAAGCTTGATCATCACAAAGCAAATTGTGCAAGGTGAAATAA |

Table S3. Primers for EcoPI *mod* and *res* genes cloning under native promoter into pBTB-2

| EcoPI_F | AGGCTTTTGACTTTCTGCTATGGAGGCATCGCTCAAAGAAGC |
| --- | --- |
| EcoPI_R | GAATGATATCAAGCTTGAATTCGTTTTATGGTAATGCGCTCTTG |
| BTBb_F | AACGAATTCAAGCTTGATATC |
| BTBb_R | CTCCATAGCAGAAAGTCAAAAG |

Table S4. Results of the λ.0 phage plaquing (EOP) on a lawn of *E. coli* cells containing genes of various RMI systems of gram-negative bacteria. Restriction and antirestriction phenotypes were estimated comparing to TG1 or TG1-RMI strain respectively. The effect of restriction and antirestriction was statistically analyzed with one-tail paired t‑test and the result is given in “restriction phenotype” and “antirestriction phenotype” columns: + observed effect, - no effect, * p-value < 0.05, ** p-value < 0.01, *** p-value < 0.001.

| Strain | Exp 1 | Exp 2 | Exp3 | Restriction phenotype (comparing with TG1) | Antirestriction phenotype (comparing with corresponding TG1-RMI strain) |
| --- | --- | --- | --- | --- | --- |
| TG1 | 1 | 1 | 1 |  |  |
| pACYC184 | 1 | 1,22 | 1,43 | - |  |
| EcoKI | 0,0001 | 0,0003 | 0,00009 | + *** |  |
| EcoKI+ArdA | 1 | 1,22 | 1,04 |  | + ** |
| EcoKI+ArdB | 0,53 | 0,45 | 0,54 |  | + ** |
| EcoKI+Ocr | 1,2 | 0,87 | 1,05 |  | +** |
| EcoAI(MS)* | 1 | 1,11 | 1,07 |  |  |
| EcoAI | 0,04 | 0,05 | 0,03 | + *** |  |
| EcoAI+ArdA | 0,15 | 0,14 | 0,13 |  | + ** |
| EcoAI+ArdB | 0,61 | 0,81 | 0,7 |  | + ** |
| EcoAI+Ocr | 1,02 | 1,15 | 1,21 |  | +** |
| EcoR124II(MS)** | 1 | 1,11 | 1,23 |  |  |
| EcoR124II | 0,0001 | 0,00014 | 0,00012 | + *** |  |
| EcoR124II+ArdB | 0,046 | 0,053 | 0,045 |  | + ** |
| EcoR124II+ArdA | 1 | 1,11 | 1,15 |  | + ** |
| EcoR124II+Ocr | 0,0028 | 0,0031 | 0,0021 |  | +* |

*,** Constructs are not shown in the main text. They were used for control, EcoAI(MS) – TG1pAM14,EcoR124II(MS) – TG1pACMS; listed in table S5.

Table S5. Supplementary plasmids.

| pAM14 | Vector pACYC184, contains genes, which encode IB RMI-system EcoAI with no R-subunit. Cm^r^. | (Kudryavtseva *et al.*, 2023) |
| --- | --- | --- |
| pACMS | Vector pACYC184, contains genes, which encode IC RMI-system EcoR124II with no R-subunit. Cm^r^. | (Weiserova *et al.*, 1993) |


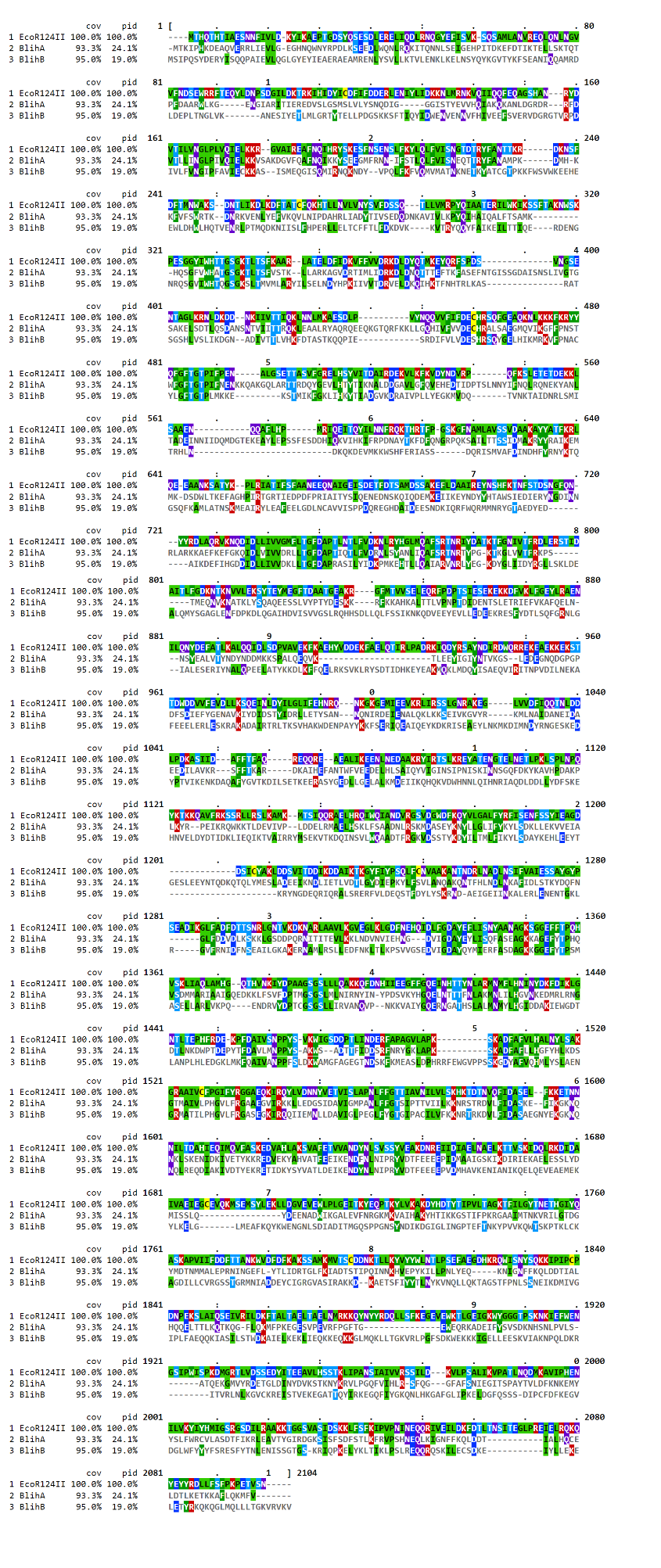


Figure S1. Aligned amino acid sequences of RM-I systems (R,M,S subunits): EcoR124II, BlihA, BlihB. The dash ( - ) sign indicates the absence of a homologous amino acid at this position in the second sequence. The Cov value presents the percent of coverage of each sequence and Pid value is the percent of identical amino acids. The alignment made with Mview web service at EMBL-EBI site (Madeira et al. 2019).

Table S6. Results of the λ.0 phage plaquing (EOP) on a lawn of *E. coli* cells containing genes of RMI systems of gram-positive bacteria comparing with EcoKI. Restriction and antirestriction phenotypes were estimated comparing to TG1 or TG1-RMI strain respectively. The effect of restriction and antirestriction was statistically analyzed with one-tail paired t‑test and the result is given in “restriction phenotype” and “antirestriction phenotype” columns: + observed effect, - no effect, * p-value < 0.05, ** p-value < 0.01, *** p-value < 0.001.

| Strain | Exp 1 | Exp 2 | Exp3 | Restriction phenotype (comparing with TG1) | Antirestriction phenotype (comparing with corresponding TG1-RMI strain) |
| --- | --- | --- | --- | --- | --- |
| TG1 | 1 | 1 | 1 |  |  |
| EcoKI | 0,0001 | 0,00009 | 0,00011 | +*** |  |
| EcoKI+ArdA | 0,35 | 0,32 | 0,31 |  | +** |
| EcoKI+ArdB | 0,43 | 0,4 | 0,45 |  | +** |
| EcoKI+Ocr | 0,31 | 0,32 | 0,3 |  | +*** |
| BlihIA | 0,001 | 0,0009 | 0,0012 | +*** |  |
| BlihIA+ArdA | 0,00271 | 0,00262 | 0,00251 |  | +** |
| BlihIA+ArdB | 0,071 | 0,074 | 0,07 |  | +*** |
| BlihIA+Ocr | 0,058 | 0,061 | 0,057 |  | +*** |
| BlihIB | 0,0036 | 0,0033 | 0,0023 | +*** |  |
| BlihIB+ArdA | 0,03 | 0,033 | 0,031 |  | +** |
| BlihIB+ArdB | 0,31 | 0,3 | 0,29 |  | +*** |
| BlihIB+Ocr | 0,029 | 0,031 | 0,028 |  | +*** |

Table S7. Results of the λ.0 phage plaquing (EOP) on a lawn of *E. coli* cells containing genes of RMIII system EcoPI or BREX defense system. Restriction and antirestriction phenotypes were estimated comparing to TG1 or TG1-RM strain respectively. The effect of restriction and antirestriction was statistically analyzed with one-tail paired t‑test and the result is given in “restriction phenotype” and “antirestriction phenotype” columns: + observed effect, - no effect, * p-value < 0.05, ** p-value < 0.01, *** p-value < 0.001.

| Strain | Exp 1 | Exp 2 | Exp3 | Restriction phenotype (comparing with TG1) | Antirestriction phenotype (comparing with corresponding TG1-RMI strain) |
| --- | --- | --- | --- | --- | --- |
| TG1 | 1 | 1 | 1 |  |  |
| EcoPI | 0,00001 | 0,00003 | 0,00001 | +*** |  |
| EcoPI+ArdA | 0,00001 | 0,00001 | 0,00001 |  | - |
| EcoPI+Ocr | 0,00001 | 0,00001 | 0,00001 |  | - |
| EcoPI+ArdB | 0,000004 | 0,00001 | 0,000006 |  | - |
| BREX | 0,04 | 0,013 | 0,027 | +*** |  |
| BREX+ArdA | 0,044 | 0,017 | 0,042 |  | - |
| BREX+Ocr | 0,414 | 0,37 | 0,33 |  | +** |
| BREX+ArdB | 0,01 | 0,03 | 0,009 |  | - |
